# When the Pediatric Gut Stops: The Pattern of Intestinal Obstruction in Children

**DOI:** 10.7759/cureus.109638

**Published:** 2026-05-25

**Authors:** Greeshma Suresh, Pranav Yadav, Surajit Das, Sarita Chowdhary, Kanika Sharma

**Affiliations:** 1 Pediatric Surgery, Institute of Medical Sciences, Banaras Hindu University, Varanasi, IND

**Keywords:** abdominal emergency, etiologies, intestinal obstruction, morbidity, pediatrics

## Abstract

Background: Intestinal obstruction is a common pediatric surgical emergency and an important cause of morbidity and mortality. This study was conducted to evaluate the current etiological pattern and outcomes of intestinal obstruction in children.

Methodology: A retrospective observational study was conducted on 213 children aged 1 month to 15 years presenting with intestinal obstruction at the Department of Pediatric Surgery, Sir Sunderlal Hospital (SSH), Varanasi, India, over 1.5 years (January 2024-June 2025). Clinical, demographic, radiological, operative, and outcome data were reviewed. Statistical analysis was performed using SPSS version 26.0 (IBM Corp., Armonk, NY). Categorical variables were expressed as frequency and percentage, and continuous variables as mean ± standard deviation. Chi-square test was used for categorical comparisons, with p < 0.05 considered statistically significant. Post hoc power analysis was performed to assess sample adequacy.

Results: The most common causes of intestinal obstruction were appendicular perforation (37, 17.37%), Meckel’s diverticulum (33, 15.49%), obstructed inguinal hernia (32, 15.02%), and intussusception (30, 14.08%). In infants (n = 44), obstructed inguinal hernia (9, 20.14%), intussusception (8, 18.18%), anorectal malformation (7, 15.91%), and duodenal obstruction (7, 15.91%) were most common. The male-to-female ratio was 139:74. Conservative management was successful in 10 (4.69%) cases, while 203 (95.30%) required surgical intervention. Overall mortality was 2 (0.94%).

Conclusion: The etiological pattern of pediatric intestinal obstruction is shifting in developing countries, with appendicular perforation, Meckel’s diverticulum, obstructed hernia, and intussusception emerging as leading causes. Delayed presentation remains the major factor contributing to adverse outcomes.

## Introduction

Intestinal obstruction is one of the most common surgical emergencies in the pediatric population and remains an important cause of morbidity and mortality worldwide [1,2]. It is characterized by partial or complete interruption of intestinal contents and may rapidly progress to bowel ischemia, perforation, sepsis, and death if not managed promptly [3,4]. Early diagnosis and timely intervention are therefore essential to improve outcomes in children [5].

Children with intestinal obstruction commonly present with abdominal distension, abdominal pain, persistent vomiting, and non-passage of stools or flatus [6]. The etiopathogenesis of obstruction varies according to age [7]. Neonatal intestinal obstruction is usually caused by congenital anomalies such as intestinal atresia, malrotation, Hirschsprung disease [8], and anorectal malformations, whereas post-neonatal intestinal obstruction is more commonly associated with acquired conditions and congenital lesions presenting later in childhood, including intussusception, obstructed inguinal hernia, appendicitis, adhesions, and volvulus [9]. Some cases of Meckel’s diverticulum, Hirschsprung disease, and anorectal malformation-associated obstruction have been included in the study despite being congenital etiologies due to their primary presentation in the post-neonatal period.

The pattern of pediatric intestinal obstruction differs between developed and developing countries due to variations in socioeconomic conditions, healthcare accessibility, nutritional status, and infection burden [10,11]. In developing countries like India, delayed presentation, poor awareness, and limited access to specialized pediatric surgical care continue to contribute significantly to morbidity and mortality [12,13]. Earlier studies from tropical countries reported worm infestation and abdominal tuberculosis as major causes of intestinal obstruction; however, recent trends suggest a gradual decline in these etiopathogeneses with changing disease patterns and improved healthcare facilities [10,14,15].

Although several studies have evaluated intestinal obstruction in children, data regarding the current etiological spectrum of post-neonatal intestinal obstruction in North India remain limited. Therefore, the present study was conducted to analyze the clinical presentation, etiological pattern, management, morbidity, and outcomes of intestinal obstruction in children presenting to a tertiary care center in Varanasi, India.

## Materials and methods

Study design and setting

This retrospective observational study was conducted in the Department of Pediatric Surgery, Institute of Medical Sciences, Sir Sunderlal Hospital (SSH), Varanasi, India. The study reviewed pediatric patients presenting with intestinal obstruction over a period of 1.5 years from January 2024 to June 2025.

Study population

Inclusion Criteria

All pediatric patients presenting with intestinal obstruction during the study period from January 2024 to June 2025 were included. A total of 213 children aged between 1 month and 15 years were enrolled using consecutive sampling. A post hoc power analysis was performed based on the obtained sample size and distribution of major etiological categories. Considering an alpha error of 0.05, a medium effect size (w = 0.30), and a total sample size of 213, the achieved statistical power was calculated to be greater than 80%, indicating adequate power to detect significant differences among the etiological groups and clinical characteristics. The post hoc power analysis supports the adequacy and reliability of the sample size used in the present study.

Exclusion Criteria

Neonates (<1 month of age), children above 15 years of age, patients not consenting to participate in the study, and children presenting with frank peritonitis and pneumoperitoneum at initial presentation were excluded.

Clinical evaluation

All patients underwent a detailed clinical examination at admission. Clinical features, including abdominal distension, abdominal pain, vomiting, constipation or non-passage of stools/flatus, fever, and bleeding per rectum, were recorded.

Investigations

Routine laboratory investigations included complete blood count, serum electrolytes, blood sugar, blood urea, and serum creatinine levels. Radiological evaluation included a plain erect abdominal X-ray in all patients, followed by ultrasonography of the abdomen to identify the probable cause and level of obstruction. Additional imaging studies were performed selectively when required.

Management protocol

Initial management consisted of intravenous fluid resuscitation, correction of electrolyte imbalance, nasogastric decompression, and administration of broad-spectrum antibiotics. Blood transfusion was administered selectively in patients with hemoglobin levels below 7 g/dL. Patients who improved with conservative management were monitored closely, while those with persistent obstruction, clinical deterioration, or suspected bowel compromise underwent surgical exploration. Out of the 213 cases, 10 patients were managed conservatively, and 203 patients required operative intervention.

Data collection

Data regarding demographic profile, clinical presentation, etiology of obstruction, operative findings, surgical procedures performed, postoperative complications, morbidity, and mortality were collected from hospital records and analyzed.

Statistical analysis

Data were entered into Microsoft Excel (Microsoft Corp., Redmond, WA) and analyzed using SPSS version 26.0 (IBM Corp., Armonk, NY). Categorical variables were expressed as frequency and percentage. Continuous variables were expressed as mean ± standard deviation wherever applicable. The Chi-square test or Fisher’s exact test was used for comparison between categorical variables. A p-value of <0.05 was considered statistically significant.

## Results

Of the 213 pediatric patients included in the study, 139 were males and 74 were females. The most frequently affected age group was 1-5 years (88, 41.31%), followed by >5 years (81, 38.03%) and <1 year (44, 20.66%). The predominant clinical features were abdominal distension (212, 99.53%), abdominal pain (203, 97.65%), failure to pass stools (205, 96.24%), and persistent vomiting (197, 92.49%), while fever (51, 23.94%) and bleeding per rectum (13, 6.1%) were less common (Table 1).

**Table 1:** Summary of clinical presentation

Clinical presentation	Number of patients	Percentage
Abdominal pain	208	97.65
Abdominal distension	212	99.53
Non-passage of stools	205	96.24
Persistent vomiting	197	92.49
Fever	51	23.94
Bleeding per rectum	13	6.1

Out of all cases, 10 (4.69%) were managed conservatively, whereas 203 (95.30%) required surgical exploration. The most common etiologies of intestinal obstruction included appendicular perforation (37, 17.37%), Meckel’s diverticulum (33, 15.49%), obstructed inguinal hernia (32, 15.02%), intussusception (30, 14.08%), and spontaneous adhesive obstruction (25, 11.75%). Less frequent causes were midgut volvulus (18, 8.45%), Hirschsprung disease (13, 6.10%), duodenal obstruction (8, 3.76%), anorectal malformation (7, 3.29%), postoperative adhesions (6, 2.81%), and septic ileus (4, 1.88%) (Table 2). The corresponding operative procedures and associated complications are detailed in Table 3. Overall mortality was 0.94% (n = 2), with one death due to bowel gangrene in midgut volvulus and the other in a case of septic ileus.

**Table 2:** Summary of observed etiopathogenesis

Etiology	Percentage (%)	Males (74)	Females (139)	Age distribution			Conservative management	Deaths
				<1 year (44)	1-5 years (88)	>5 years (81)		
Appendicular perforation	37 (17.37%)	28	9	-	24	13	-	-
Meckel’s diverticulum	33 (15.49%)	17	16	4	13	16		
Obstructed inguinal hernia	32 (15.02%)	31	1	9	19	4		
Intussusception	30 (14.08%)	19	11	8	3	19		
Spontaneous adhesive obstruction	25 (11.75%)	11	14	1	11	13	1	
Midgut volvulus	18 (8.45%)	6	12	-	5	13	-	1
Hirschsprung disease	13 (6.10%)	11	2	4	6	3	5	-
Duodenal obstruction	8 (3.76%)	1	7	7	1	-	-	
Anorectal malformation	7 (3.29%)	5	2	7	-	-		
Postoperative adhesions	6 (2.81%)	6	-	4	2	-		
Septic ileus	4 (1.88%)	4	-	-	4	-	4	1

**Table 3:** Summary of procedures and associated morbidity SSI: surgical site infection, SAIO: sub-acute intestinal obstruction

Procedure	Number of patients operated on (n = 203)	Percentage	Complications
Bowel resection and anastomosis	59	29.06%	SSI: 3
Colostomy	13	6.40%	Stoma prolapse: 1
Reduction of intussusception	6	2.96%	-
Herniotomy	29	14.28%	-
Ladd’s procedure	17	8.37%	-
Adhesiolysis	31	15.27%	SAIO: 1
Anoplasty	4	1.97%	-
Appendicectomy	36	17.73%	SSI: 1
Duodenoduodenostomy	8	3.94%	-

## Discussion

The incidence of various causes of obstruction varies with age and demographic profile. The various causes of obstruction in the post-neonatal period in our study have been summarized in Table 2. In our study, the most common cause of obstruction was noted to be appendicular perforation, where the most common age group was 1-5 years [16,17]. This was followed by Meckel’s diverticulum-associated causes of obstruction, which included Meckel’s band, Meckel’s diverticulum perforation, and intussusception with Meckel’s diverticulum as the lead point [9,18]. Although Meckel’s is a congenital entity, the cases included here had presentation after the neonatal period. The most common age group was above 5 years, closely followed by the 1-5 years age group. This was followed by an obstructed inguinal hernia, most commonly presenting between 1 and 5 years [19]. Intussusception was noted to be the subsequent most common cause of obstruction, of which the most common was ileocolic, followed by ileo-ileal intussusception [20]. The next common cause of obstruction was spontaneous adhesive obstruction (including abdominal tuberculosis), which was slightly more common in females and in the age group above 5 years [21].

Midgut volvulus was also a notable entity with associated morbidity and mortality due to delayed presentation [22]. Hirschsprung disease was noted more commonly between 1 and 5 years, and were initially conservatively managed in the majority of cases due to the severe enterocolitis at presentation [23]. Other notable causes of obstruction were duodenal obstruction (including duodenal web, annular pancreas, and SMA syndrome), anorectal malformations (anal stenosis, cloacal anomalies, and vestibular anus), postoperative adhesions, and septic ileus [24,25]. There were two deaths, one due to severe sepsis among the cases of septic ileus and one in malrotation due to near-total bowel gangrene at presentation [26,27].

We had excluded cases of neonatal obstruction as there is a large bulk of cases in this individual category, mandating an independent study in this age group. We had also excluded those presenting with generalized peritonitis and pneumoperitoneum (on X-ray) at presentation and also the causes of abdominal trauma [19,28]. However, appendicular perforation in all cases included in the study had presented with features of intestinal obstruction without peritonitis or pneumoperitoneum and, hence, were included in the study. We have noted that the causes of obstruction due to worm impaction and abdominal Kochs have declined as compared to previous studies at similar demographic distribution. Also, mortality rates have decreased, probably due to earlier medical attention and advancements in medical care [29,30].

Strengths and limitations of the study

This study includes a relatively large sample size from a tertiary care center and provides updated data on the etiological spectrum of post-neonatal intestinal obstruction in children. It highlights changing trends in causes, includes a broad pediatric age group, and offers a detailed analysis of clinical presentation, management, and outcomes, with an adequate post hoc statistical power (>80%). However, it is limited by its retrospective single-center design, exclusion of neonatal cases, and lack of long-term follow-up. Incomplete assessment of socioeconomic factors and non-uniform use of advanced imaging further limit generalizability.

## Conclusions

The most common causes of intestinal obstruction in post-neonatal children are appendicular perforation, Meckel’s diverticulum-related causes of obstruction, obstructed inguinal hernia, and intussusception, followed by other causes. The pattern of intestinal obstruction among the pediatric population is similar in developing countries. The probable reasons for the associated morbidity could be poor socioeconomic status, illiteracy, lack of awareness, and delay in seeking medical assistance, demanding further studies and social and medical policymaking at the administrative levels.

## References

[1] Alashaby SS, Gilan WM, Al-Absy TA, Al-Magedi AA (2026). Etiology, clinical profile, management, and outcomes of intestinal obstruction in a resource-limited setting: a prospective study. Sci Rep.

[2] Tamirat A, Nigussie J, Biset G (2025). Surgical outcome of pediatric intestinal obstruction in Amhara comprehensive specialized hospitals, September 2024. BMC Surg.

[3] Bala M, Catena F, Kashuk J (2022). Acute mesenteric ischemia: updated guidelines of the World Society of Emergency Surgery. World J Emerg Surg.

[4] Stancu B, Chira A, Coman HF, Mihaileanu FV, Ciocan R, Gherman CD, Andercou OA (2024). Intestinal obstruction as initial presentation of idiopathic portal and mesenteric venous thrombosis: diagnosis, management, and literature review. Diagnostics (Basel).

[5] Adeoye MA, Sulaimon JT, Iliasu SF, Fawole RO (2025). Evaluating the efficacy of early intervention programs for children with developmental disorders. Idarotuna.

[6] Suraj KC, Yogi TN, Rauniyar K (2025). Duodenal ulcer perforation peritonitis as a cause of acute abdomen in pediatric population: a rare case report and review of literature. Ann Med Surg (Lond).

[7] Wang Z, Lin J, Liang L (2025). Global, regional, and national burden of chronic obstructive pulmonary disease and its attributable risk factors from 1990 to 2021: an analysis for the Global Burden of Disease Study 2021. Respir Res.

[8] Kadhim MJ (2025). Clinical profile and outcomes of neonates with intestinal obstruction: a retrospective study from Karami hospital. Eur J Med Health Res.

[9] Zanchetta M, Inversini D, Pappalardo V (2023). Meckel’s diverticulum causing ileal volvulus and peritonitis after a recent appendectomy: a case report and literature review-we should likely resect an incidental MD. Life (Basel).

[10] Adams NL, Rose TC, Hawker J (2018). Relationship between socioeconomic status and gastrointestinal infections in developed countries: a systematic review and meta-analysis. PLoS One.

[11] Twahirwa I, Ndayiragije C, Nyundo M, Rickard J, Ntaganda E (2022). Pediatric intestinal obstruction: analysis of etiologies and factors influencing short-term outcomes in Rwanda. World J Pediatr Surg.

[12] Vuddemarry M, Zadey S, Sp A, Ghosh D, Zheleva B (2026). Care pathways for pediatric surgical conditions. World J Pediatr Surg.

[13] Ahmed MM, Oweidat M, Okesanya OJ, Alaswad M, Abdelbar SM, Gill P, Alsabri M (2025). Barriers to pediatric emergency care in low-resource settings: a narrative review. Sage Open Pediatr.

[14] Ghazy RM, Alshaikhi SA, Assiri HA, Almozaini AA, Alhazmi AF, Elhasaneen HE, Abdo SM (2025). Tropical diseases and the gastrointestinal tract: an overlooked connection. Front Trop Dis.

[15] Shiva S, Kumar S, Singh P, Kumar S, Kumar S (2025). The silent menace: worm bezoar causing acute intestinal obstruction. J Family Med Prim Care.

[16] Pal GC, Badhan RE, Chowdhury MT, Moushumi RS (2025). Appendix perforation: contributing risk factors. Int Surg J.

[17] Sagir IH (2026). Risk factors for perforation in acute appendicitis. Al-Qadisiyah Med J.

[18] Hernández JD, Valencia G, Girón F (2023). Meckel's diverticulum: analysis of 27 cases in an adult population. Front Surg.

[19] Morini F, Dreuning KM, Janssen Lok MJ (2022). Surgical management of pediatric inguinal hernia: a systematic review and guideline from the European Pediatric Surgeons’ Association Evidence and Guideline Committee. Eur J Pediatr Surg.

[20] Mușat F, Păduraru DN, Bolocan A, Constantinescu A, Ion D, Andronic O (2023). Endometriosis as an uncommon cause of intestinal obstruction-a comprehensive literature review. J Clin Med.

[21] Tenginkai P, K SN, Nagaraj P (2020). Etiological factors, various modes of clinical presentation of acute intestinal obstruction. Int J Surg Sci.

[22] Dehaini H, Nasser Eldine R, Doughan S, Khalifeh M, Khasawneh H, Hussain H, Sbaity E (2020). Presentation of intestinal malrotation and midgut volvulus in adults: case report & literature review. Int J Surg Case Rep.

[23] Roorda D, Oosterlaan J, van Heurn E, Derikx JP (2021). Risk factors for enterocolitis in patients with Hirschsprung disease: a retrospective observational study. J Pediatr Surg.

[24] Taşdemir Ü, Demirci O (2025). Clinical analysis of congenital duodenal obstruction and the role of annular pancreas. Medicina (Kaunas).

[25] Mohamed SS, Mead AO, Nur AO (2025). Annular pancreas in a 13-year-old boy: a delayed clinical presentation of a congenital anomaly highlighting challenges of diagnosis. Int Med Case Rep J.

[26] Jaiswal R, Jaiswal SA, Husukale HD, Bendale H, Chavan SR (2024). From volvulus to short bowel syndrome: the impact of extensive gangrene. Cureus.

[27] Lamprinou Z, Kanna E, Skondras I, Sfakiotaki R, Mene J, Achilleos O (2024). Internal hernia as cause of acute abdomen in a preterm neonate: when necrotizing enterocolitis is not the culprit. Arch Clin Cases.

[28] Liu Y, Kang Q, Liu G (2023). Diagnosis, risk factors, and treatment of pediatric benign pneumoperitoneum: a single-center retrospective study. Pediatr Discov.

[29] Bhandari R, Chamlagain R, Sutanto E (2022). Intestinal perforation due to adult tapeworm of Taenia: a case report and review of the literature. Clin Med Insights Case Rep.

[30] Asfaw BA, Yigzaw KT, Bhatta OP, Asfaw YA, Fenta BA, Wassie MT (2025). Gangrenous small bowel volvulus due to ascariasis in a 9-year-old female: a case report. J Pediatr Surg Case Rep.

